# Removing of the Sulfur Compounds by Impregnated Polypropylene Fibers with Silver Nanoparticles-Cellulose Derivatives for Air Odor Correction

**DOI:** 10.3390/membranes11040256

**Published:** 2021-04-01

**Authors:** Aurelia Cristina Nechifor, Simona Cotorcea, Constantin Bungău, Paul Constantin Albu, Dumitru Pașcu, Ovidiu Oprea, Alexandra Raluca Grosu, Andreia Pîrțac, Gheorghe Nechifor

**Affiliations:** 1 Department of Analytical Chemistry and Environmental Engineering, University Politehnica of Bucharest, 1-7 Gheorghe Polizu St., 011061 Bucharest, Romania; aureliacristinanechifor@gmail.com (A.C.N.); cotorceasimona@yahoo.com (S.C.); dd.pascu@yahoo.com (D.P.); andreia.pascu@yahoo.ro (A.P.); ghnechifor@gmail.com (G.N.); 2 Department of Engineering and Management, Faculty of Management and Technological Engineering, University of Oradea, 410087 Oradea, Romania; bungau@uoradea.ro; 3 Department of Radioisotopes and Radiation Metrology, IFIN Horia Hulubei, 30 Reactorului St., 023465 Magurele, Romania; paulalbu@gmail.com; 4 Department of Inorganic Chemistry, Physical Chemistry and Electrochemistry, University Politehnica of Bucharest, 1-7 Gheorghe Polizu St., 011061 Bucharest, Romania; ovidiu73@yahoo.com

**Keywords:** sulfur compounds, polypropylene fibers, silver nanoparticles, cellulose, membranes, air odor correction, hydrogen sulfide, ethanethiol, membrane processes

## Abstract

The unpleasant odor that appears in the industrial and adjacent waste processing areas is a permanent concern for the protection of the environment and, especially, for the quality of life. Among the many variants for removing substance traces, which give an unpleasant smell to the air, membrane-based methods or techniques are viable options. Their advantages consist of installation simplicity and scaling possibility, selectivity; moreover, the flows of odorous substances are direct, automation is complete by accessible operating parameters (pH, temperature, ionic strength), and the operation costs are low. The paper presents the process of obtaining membranes from cellulosic derivatives containing silver nanoparticles, using accessible raw materials (namely motion picture films from abandoned archives). The technique used for membrane preparation was the immersion precipitation for phase inversion of cellulosic polymer solutions in methylene chloride: methanol, 2:1 volume. The membranes obtained were morphologically and structurally characterized by scanning electron microscopy (SEM) and high resolution SEM (HR SEM), energy dispersive X-ray analysis (EDAX), Fourier transform infrared spectrometry (FTIR), thermal analysis (TG, ATD). Then, the membrane performance process (extraction efficiency and species flux) was determined using hydrogen sulfide (H_2_S) and ethanethiol (C_2_H_5_SH) as target substances.

## 1. Introduction

The international community’s concern for air quality began with the industrial development, but the link to public health only emerged after major accidents consequently contributing to the loss of human lives and amplification of chronic diseases [1,2,3].

Preventing and combating air pollution has led to the imperative need to develop and implement effective measures on a large scale, including [4,5,6,7]:

- Relocation of polluting production units outside the localities;
- Giving up to polluting technologies;
- Improving production processes;
- Mandatory control of pollutant emissions and regulations regarding the allowed concentration levels;
- Reducing of greenhouse gas emissions;
- Promoting new technologies, with a tendency to allow only the implementation of clean or ecological/green technologies, which are more friendly to the environment;
- Use of recyclable materials, etc.

In many economically advanced countries polluting production processes have been abandoned, and many of the necessary materials (obtained through polluting technologies) are imported (i.e., chemical and metallurgical products, leather and textiles, construction materials, and often even processed food and energy) [8,9,10].

However, despite the progress made, complex equipment, installations, and production units with high impact on air quality remained in operation and are common [11].

In this ocean of problems, regulations have been focused on imposing reductions for the emissions of organic or inorganic substances that are known to be toxic, carcinogenic, or dangerous, by prohibiting their usage or by imposing restrictions and drastic limits [12].

Without being neglected, the unpleasant smell of the community air remained insufficiently regulated and difficult to monitor, the main problem being related to the extremely low concentrations in which some substances generate it [12,13].

**Figure 1:**
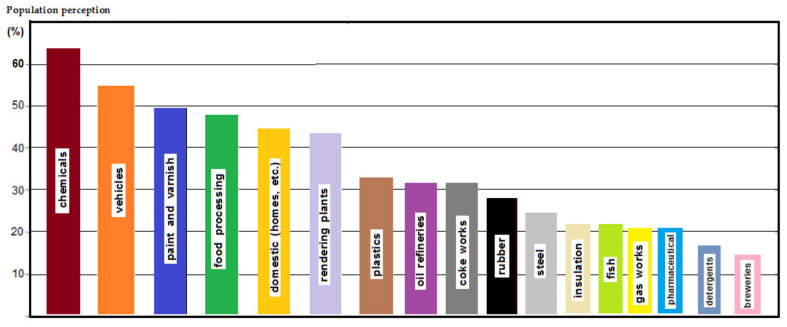
Perception of the population about the foul-smelling sources.

Yet it is interesting that, since the middle of the last century, the population was aware of this type of pollution sources (chemicals, food processing, plastics, gas emission, cleaning products, etc.), a statistical study indicating this perception is presented in Figure 1 [14].

In these circumstances, the processes and technologies for preventing and combating unpleasant odors have constantly evolved [15], the best-known methods involving thermal [16], biological [17], physical and chemical [18,19], catalytic [20], electro-physical [21], or membrane processes [22].

A well-established and effective way to remove odors uses adsorption of aerosol micro-particles followed by their retention on filters; electrical precipitators, sonic flocculators, and cyclone scrubbers are also available for removing these particles [23].

**Figure 2:**
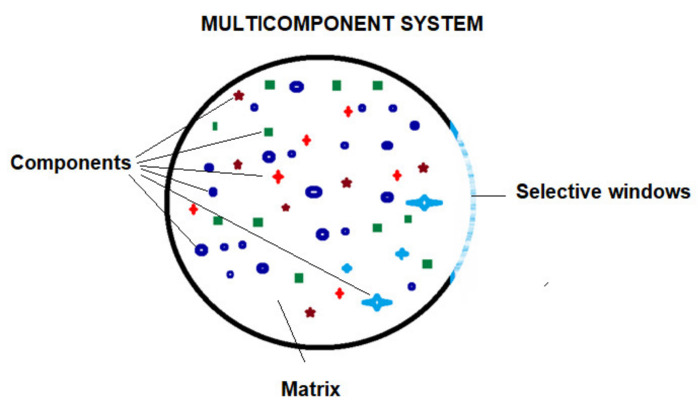
Multicomponent system bordered by a selective window: ions, small molecules, macromolecules, nanoparticles, micro-particles, microorganisms, and viruses, suspended particles.

If we focus on membrane processes, it can be stated that the membrane is a window of a multi-component system (Figure 2), with selective permeability for chemical species of the system. Regardless of the nature or state of aggregation of the membrane material, the defining characteristic of the membrane is its semi/selective permeability [24,25,26,27]. This means that the chemical species that make up the system can come out only directed or controlled, through the window (membrane).

The membrane that lets a chemical species out of the system (it is permeable to it) will not be able to stop these chemical species from returning inside if their activity (or concentration) outside the system is high.

To optimize the separation processes, the membranes must meet certain requirements regarding selectivity; flow (permeability); and chemical, thermal, and mechanical stability for various applications [28].

Not all these properties can be fulfilled simultaneously. Therefore, for separation in optimal conditions, the membranes and the corresponding technique are designed to separate the components from a certain mixture, starting from its physical/chemical properties and from the imposed requirements [28,29]. In the mentioned context, the design of the membranes is done by acting on the membrane material, which must satisfy most of the imposed requirements [30]; further, engineers work on the system separation process considering a multitude of operating parameters as temperature, pH, ionic strength, and addition of surfactants or nanoparticles [31,32].

In the last fifty years, membranes and membrane processes have evolved from laboratory-scale to industrial installations, having an increased economic and commercial importance [33,34] and being also closely connected to human health (the use of membranes to remove harmful components from the air [35,36]).

Currently, the development of membrane processes is at an intermediate stage: the first generation of membrane processes (microfiltration, ultrafiltration, reverse osmosis, electro-dialysis, membrane electrolysis) being in the stage of optimization and expansion of applications, while second-generation membrane processes (nanofiltration, gas separation, pervaporation, membrane distillation, liquid membranes) are in continuous evaluation not being promoted at an industrial scale yet [34]. Among the problems that have determined the exponential development of membrane processes are those related to environmental protection, the fact that technologies based on membranes and membrane separation techniques are ecological being well known [33]. Consequently, the design and operation of membrane processes have grown considerably [37].

The problem approached in this study is of increased difficulty due to the compositional complexity of the gaseous systems that are at the origin of the bad smell [38,39,40,41,42].

Usually, some substances are associated with a known (common) smell: hydrogen sulfide (H_2_S) with rotten eggs; methyl mercaptan (CH_3_SH) and dimethyl disulfide (CH_3_SSCH_3_) with pungent; dimethyl sulfide (CH_3_SCH_3_) with unpleasant sweet; carbon disulfide (CS_2_) with lightly pungent; ammonia (NH_3_) with pleasantly sweet; dimethylamine ((CH_3_)_2_NH) with fishy, ammoniacal smell; allyl mercaptan (CH_2_=CHCH_2_ SH) and allyl methyl sulfide (CH_2_=CHCH_2_ SCH_3_) with garlic-like smell [43,44,45].

Thus, in order to determine which are the different gaseous chemicals that produce unpleasant odors, it is necessary to start from the components that appear in the most diverse cases (Table 1) [44,45,46,47,48,49].

Of course, such sources of bad-smelling substances can be found in almost every locality or near it, but their effect is felt little and quite seldom.

**Figure 3:**
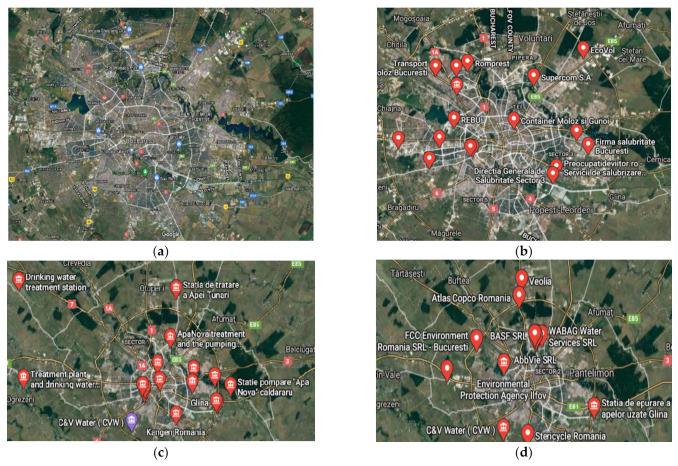
The Bucharest metropolitan area and its foul-smelling gases’ pollution sources: (**a**) the metropolitan area; (**b**) the location of main municipal waste processing and recovery centers; (**c**) locations of water treatment plants; (**d**) agencies and associations involved in environmental issues.

However, this is not the case in the Romanian capital city, Bucharest, which, together with the metropolitan area, covers an area of approximately 250 km^2^, has nearly 3 million inhabitants, and provides 20–25% of the economic capacity of the country. Among the numerous air pollution problems (due to almost 3 million vehicles/day, either local or transiting; factories that process construction materials; thermo-electric power plants—either gas- or tar-based; both large and small meat, fish, or milk processing unities), this city also deals with the special case of foul-smelling gases generation, a fact leading to the unfortunate situation that renders Bucharest as the world capital of air pollution (according to statistics from December 2020) [50,51]. For the last cause listed above, identified as mainly responsible are the improperly treated water, plants, municipal waste storage, and waste processing centers (Figure 3).

The distribution of pollution sources determines the appearance of foul-smelling gases within the metropolitan area regardless of wind direction, this being more pronounced in spring and autumn. Although the composition of pollution sources in Bucharest is varied, the foul-smelling gases are mainly hydrogen sulfide and various mercaptans. Of these target substances, hydrogen sulfide and ethanethiol were selected for the present study.

It is understandable why the pollutant must be removed from the liquid or gaseous source that generates it, because after the dispersion its elimination is either inefficient (due to dilution) or very expensive. Therefore, the use of accessible, efficient, and low-cost materials (even if they are residues of other units) is desirable.

Without being exhaustive, it can be stated that the bad smell is generated by hydrogen sulfide and its organic derivatives (especially alkylates). In fact, the characterization of hydrogen sulfide as a colorless gas with a specific bad odor that stems in most cases from the decomposition of organic matter is known, and its limits in the air are strictly regulated [43,52]. It is also a by-product of various industrial processes and is an important cause of work-related sudden death [53].

Specific treatments that can be applied may fall in the classical chemical reactions (neutralization, acidification, alkalization, oxidation, reduction, hydrolysis, polymerization, etc.) [54,55].

The interaction of membrane processes with various hydrogen sulfide removal technologies from gaseous or liquid mixtures was predictable because membrane methods and techniques are characterized by simple and scalable installations, selectivity and flows are guided by accessible operating parameters (pH, temperature, redox potential, ionic strength), automation is complete, and operating costs are low (Table 2).

However, in the performed studies (Table 2) the removal of hydrogen sulfide was considered, first of all, in order to capitalize on the other components of the feed phase. At the same time, the costs of membrane materials were not evaluated, the research being focused on their selectivity.

The involvement of membranes in the treatment of effluents with polluting potential, in particular bad smell generators, requires the approach of quantitatively accessible materials, technologically and economically efficient, so that they can be applied to an industrial scale.

In this paper, we aimed to study the removal of hydrogen sulfide or ethanethiol (as tasks of the odor-generating substances) through/with the help of membranes obtained from cellulosic derivatives containing silver nanoparticles, using accessible raw material (namely motion picture films from abandoned archives). This objective was considered important in order to correct the smell of effluents resulting from the processing of municipal waste.

## 2. Materials and Methods

### 2.1. Materials

#### 2.1.1. Chemicals

The materials used in the present work were of analytical purity. They were purchased from Merck (Merck KGaA, Darmstadt, Germany)—sodium sulfide (Na_2_S; 78.0452 g/mol (anhydrous)), sodium hydroxide, and hydrochloric acid; and from Sigma-Aldrich (Merck KGaA, Darmstadt, Germany)—ethanethiol (C_2_H_5_SH; 62.13404 g/mol^−1^, density 0.8617 g/cm^−3^, solubility in water 0.7% (20 °C)), methylene chloride, methanol, silver nitrate.

The purified water, characterized by 18.2 µS/cm conductivity, was obtained using a RO Millipore system (MilliQ^R^ Direct 8 RO Water Purification System, Merck, Darmstadt, Germany).

The tubular dialysis membranes were from Visking (Medicell Membranes Ltd., London, UK). MQuant® sulfide test (Merck Millipore, Darmstadt, Germany), sulfide test photometric, Spectroquant^®^ (Merck KGaA, Darmstadt, Germany).

#### 2.1.2. Membrane Support

The hollow fibers polypropylene support membranes (PPSM) were provided by GOST Ltd., Perugia, Italy (Table 3). The average flow of permeate for pure water was (10–15 L/m^2^ h), at operation pressure (0.1–0.4 bar), on microfiltration processes [72,73].

### 2.2. Impregnated Ag-Cellulose Acetate Polypropylene Membrane Preparation (Ag-Cell-Ac-PPM)

#### 2.2.1. Obtaining Ag-Cellulose Acetate Recovered from Film Solutions

The used (degraded, abandoned) motion films, based on cellulose acetate, of 70 mm/65 mm, 35 mm, 32 mm (2 × 16 mm^2^), 16 mm, and 8 mm from the Romanian film archives were cut to millimeter dimensions and then disintegrated in the colloidal mill to micron dimensions.

After being washed twice with a 0.01 mol/L hydrochloric acid solution, they underwent dialysis with pure water until they were chlorine-free (test with a 0.1 mol/L AgNO_3_ solution), then were dried in a vacuum oven (45 °C), after which they were dissolved (20 g/L) in a mixture of methylene chloride and methanol, at a 2:1 volumetric ratio, in an ultrasonic bath for 10 h.

#### 2.2.2. Obtaining Ag-Cellulose Acetate Impregnated on Polypropylene Fibers Membranes (Ag-Cell-Ac-PPM)

**Figure 4:**
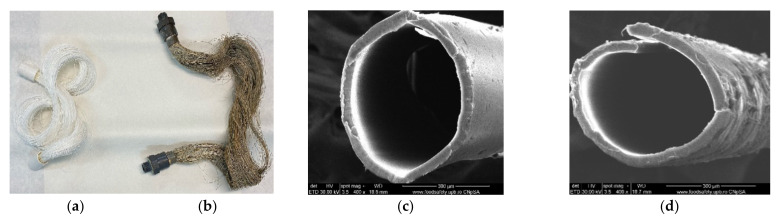
The presentation of (**a**) the polypropylene support membrane (PPSM); and (**b**) Ag-cellulose acetate membranes on PPSM (Ag-Cell-Ac-PPM); (**c**) Scanning Electron microscopy (SEM) image of PPSM; and (**d**) SEM image of Ag-Cell-Ac-PPM.

The commercially available capillary hollow fibers support (PPSM) was made from surface-modified polypropylene (PP) to provide optimal porosity (Table 1) [74]. For impregnation of the capillary bundle, they were placed in a U-shape. Then the crevices were immersed for 4 h in a 2 L cylindrical vessel containing the 1.5 L prepared 2%, 4%, and 6% Ag-cellulose acetate solution in methylene chloride: methanol solution at a volume ratio of 2:1. The membranes were removed and suspended for 24 h in a niche with laminar airflow to remove the polymer solution from outside the capillary walls (Figure 4).

The volume or amount of polymer solution in the membrane walls could be readily determined by the gravimetric method after weighing the initial membrane and the impregnated membrane.

The polypropylene support fibers, impregnated with the cellulose acetate solution, were placed suspended in a vacuum oven and evaporated at 45 °C for 4 h. After this interval, they were placed in a desiccator for cooling and then were weighed. The operations were repeated every 20 min until a constant mass was obtained. 

The percentage of the inclusion membrane (IM) was calculated using the following equation [73,74]:

(1) $$IM\left(\%\right)=\frac{\left(W_{t}-W_{i}\right)}{W_{t}}\times 100$$

where: *Wi* and *Wt* are the masses of the PPMS and the Ag-Cell-Ac-PPM membranes, respectively.

### 2.3. Permeation Procedures

**Figure 5:**
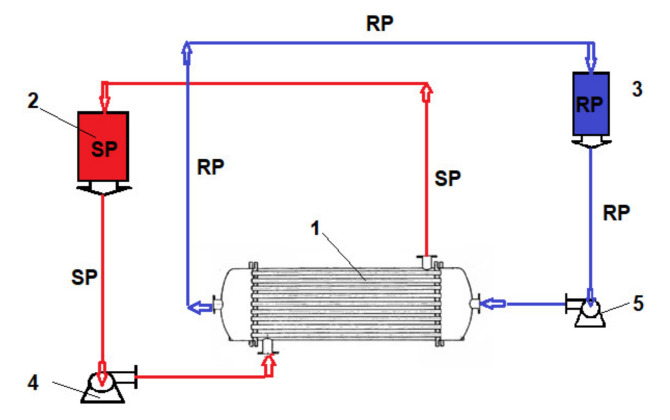
Operation scheme presentation with the pertraction module: SP—source phase; RS—receiving phase. (1) Hollow fiber pertraction module; (2) SP reservoirs; (3) RP reservoirs; (4) SP pump; (5) RP pump.

In the source phase (SP), the synthetic solution of the considered chemical species (hydrogen sulfide or mercaptans) with a concentration of 5–150 mg/L Na_2_S and pH = 5, or a 2–35 mg/L ethanethiol and pH = 5 was introduced in the installation (Figure 5) with a hollow fiber bundle that assured an effective mass transfer surface of 1.0 m^2^. The receiving phase (RP) was formed by a 0.01 mol/L sodium hydroxide solution. Three aqueous samples of 1 mL from the SP or the RP considered chemical species synthesized solutions were periodically spectrophotometrically analyzed (CamSpec Spectrophotometer, Garforth, Leeds, UK) [75,76,77,78]. The operation was performed with the receiving phase through capillaries and the source phase outside the capillaries (Figure 5).

The fluxes from the source phase [79] were determined against the measured permeate mass within a determined time range by applying the following equation:

(2) $$J=\frac{M}{S\times t}\left(\mathrm{mg}/m^{2}h\right)$$

where: *M*—permeate mass (g), *S*—effective surface of the membrane (m^2^), *t*—time (h) necessary to collect the permeate volume.

The extraction efficiency (EE%) for the species of interest using the concentration of the solutions [80] was calculated as follows:

(3) $$EE\left(\%\right)=\frac{\left(c_{0}-c_{f}\right)}{c_{0}}\times 100$$

where: *c_f_*—final concentration of the solute (considered chemical species), *c_o_*—initial concentration of the solute (considered chemical species).

The same extraction efficiency can also be computed based upon the absorbance of the solutions, as in:

(4) $$EE\left(\%\right)=\frac{\left(A_{0}-A_{s}\right)}{A_{0}}\times 100$$

where: *A*_0_—initial absorbance of the sample solution, *A_s_*—current absorbance of the sample.

The measurements were independently validated using a gas detector Oldham (MX 21 Plus Multigas, Arras, France) equipped with electrochemical sensors or an H_2_S Model 3000RS Analyzer (MultiLab LLC, Bucharest, Romania) [81,82].

### 2.4. Equipment

The microscopy studies, scanning electron microscope (SEM) and high resolution SEM (HR SEM), were performed using a Hitachi S4500 system (Hitachi High-Technologies Europe GmbH, Mannheim, Germany).

Thermal characterizations were performed using a Netzsch Thermal Analyzer (NETZSCH-Gerätebau GmbH, Selb, Germany). The thermal analysis was run in a nitrogen atmosphere at a 10 °C/min heating rate, from room temperature (25 °C) up to 900 °C.

Spectroscopy Bruker Tensor 27 FTIR with a Diamond Attenuated Total Reflection-ATR (Bruker) was used to study the interactions between the chemicals used in the membranes developed. FTIR analysis was recorded in the range of 500 to 4000 cm^−1^.

UV-VIS analysis was performed on a Spectrometer CamSpec M550 (Spectronic CamSpec Ltd., Leeds, UK). 

Other devices used were as follows: Ball Mill Retch (VIOLA—Shimadzu, Bucharest, Romania), vacuum oven (VIOLA—Shimadzu, Bucharest, Romania).

## 3. Results 

### 3.1. Scanning Electron Microscopy Studies (SEM and HFSEM and EDAX)

#### 3.1.1. Movie Films (Ag-Cellulose Acetate) 

**Figure 6:**
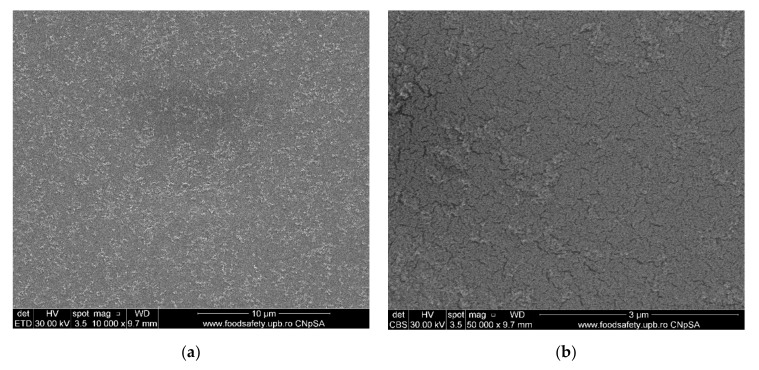

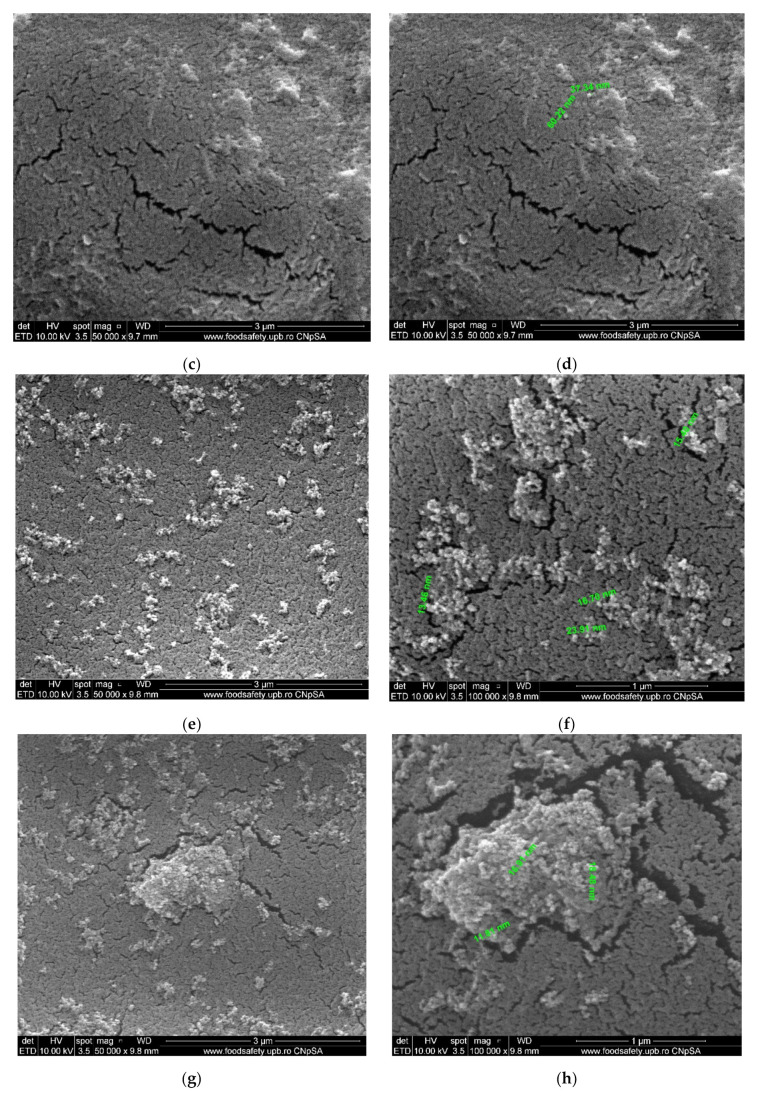

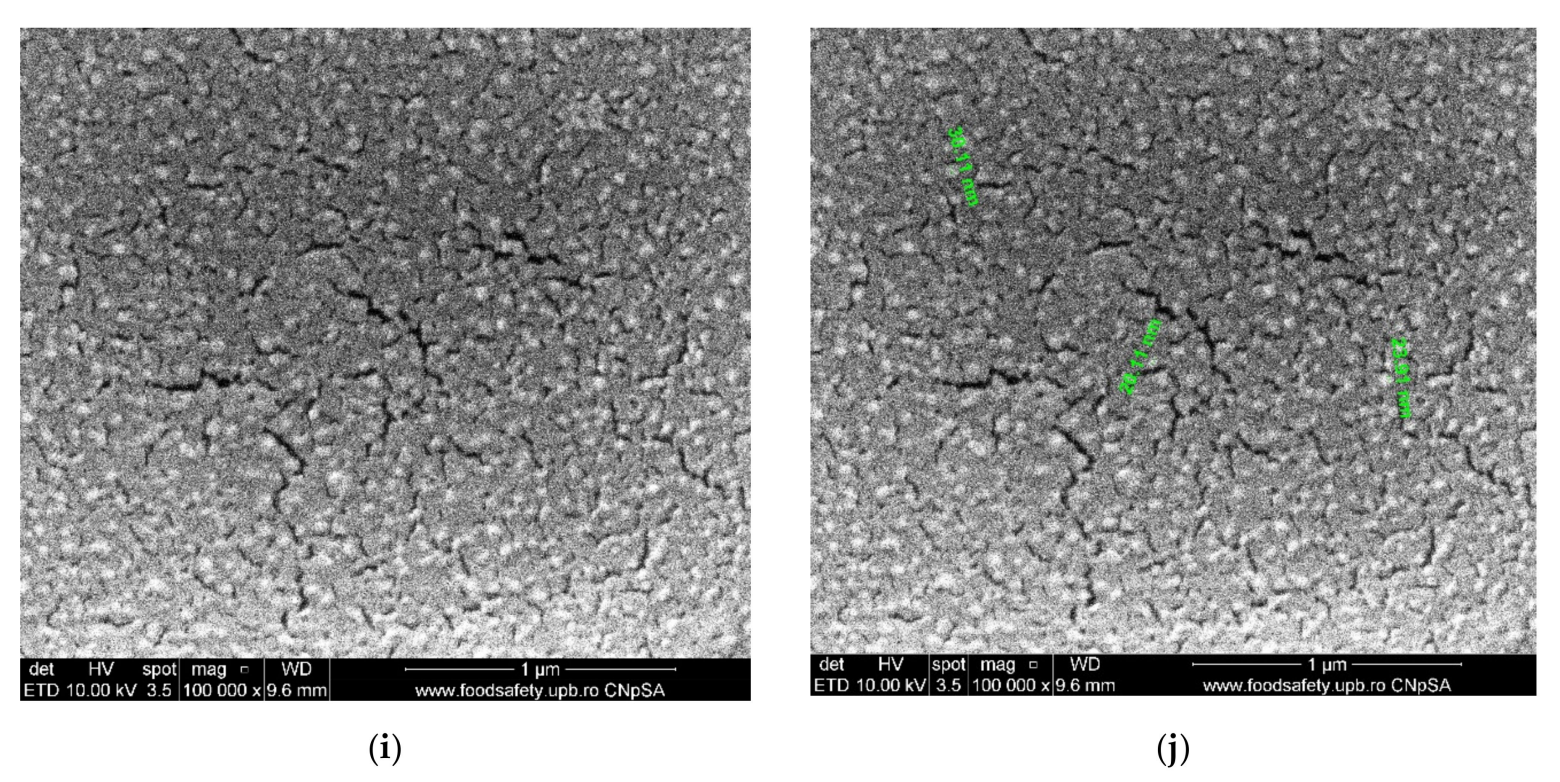
Morphology of movie film surfaces: (**a**) evenly distributed silver nanoparticles on film surface; (**b**) detail from (**a**); (**c**) micro-cracks and agglomerations of Ag nanoparticles; (**d**) detail and measurement; (**e**) Ag nanoparticles on film surface; (**f**) detail with measurable irregular micrometric lengths; (**g**) Ag nanoparticles on film surface; (**h**) measurements of micro-cracks and Ag agglomerations; (**i**) degraded film samples; (**j**) measurements of the details.

The surface morphology of the samples (movie films) was analyzed using a scanning electron microscope (SEM). All samples were properly dried prior to the analysis and were sufficiently coated with a sputtered gold layer of 400 Å (Figure 6).

#### 3.1.2. Membrane Characterization

**Figure 7:**
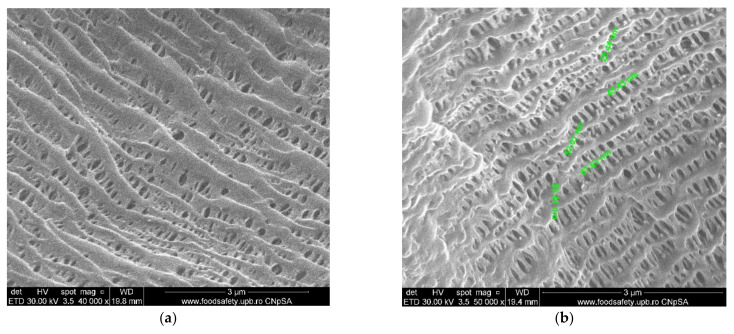

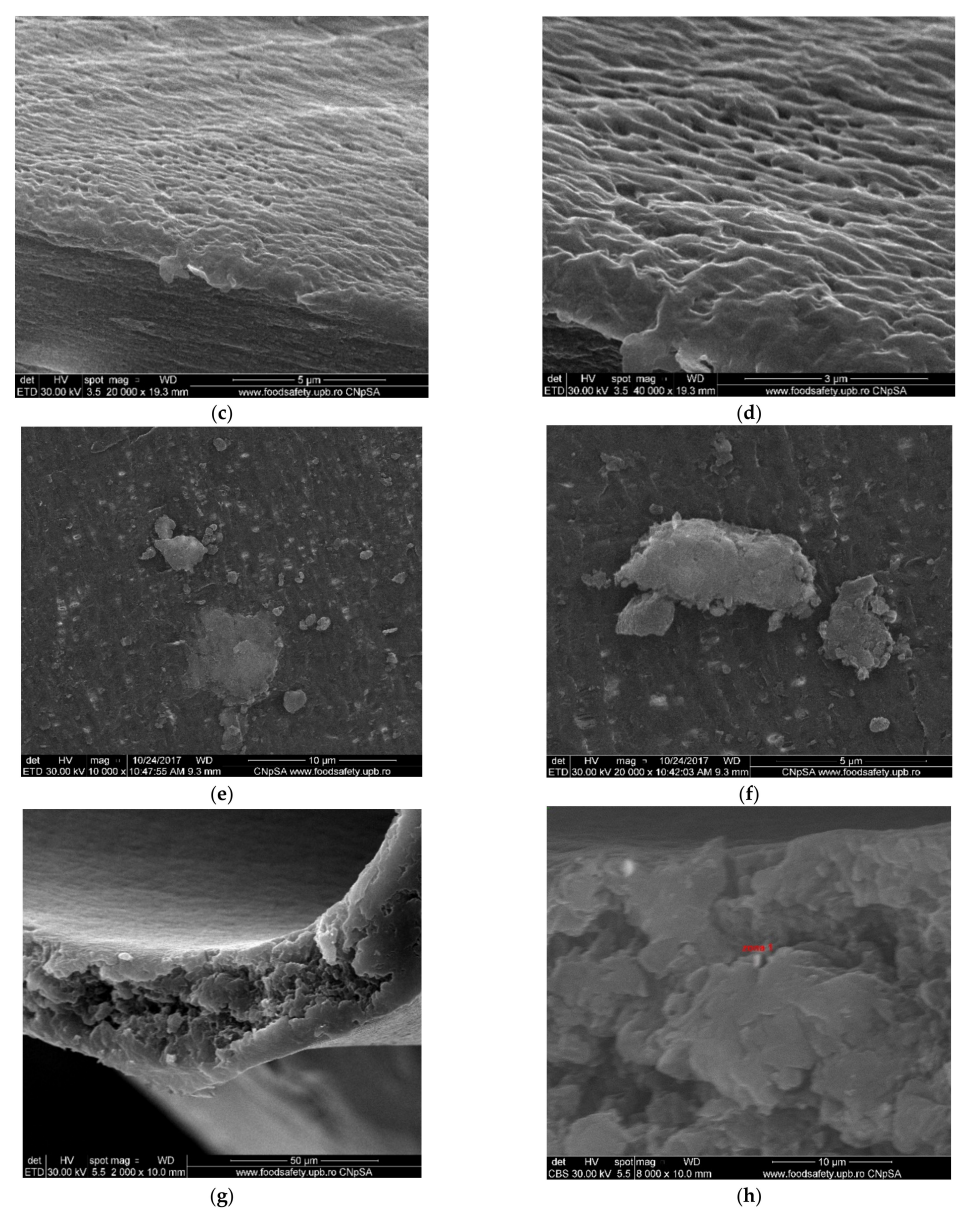
SEM morphology of the membranes: (**a**) membrane support (PPMS); (**b**) membrane support (PPMS) detail; (**c**) impregnated membrane (Ag-Cell-Ac-PPM); (**d**) impregnated membrane (Ag-Cell-Ac-PPM) detail; (**e**) processed impregnated membrane (Ag-Cell-Ac-PPM); (**f**) processed impregnated membrane (Ag-Cell-Ac-PPM) detail; (**g**) cross-section on Ag-Cell-Ac-PPM; (**h**) detail of the cross-section on Ag-Cell-Ac-PPM with the evidenced Energy Dispersive X-Ray Analysis (EDX) zone.

**Figure 8:**
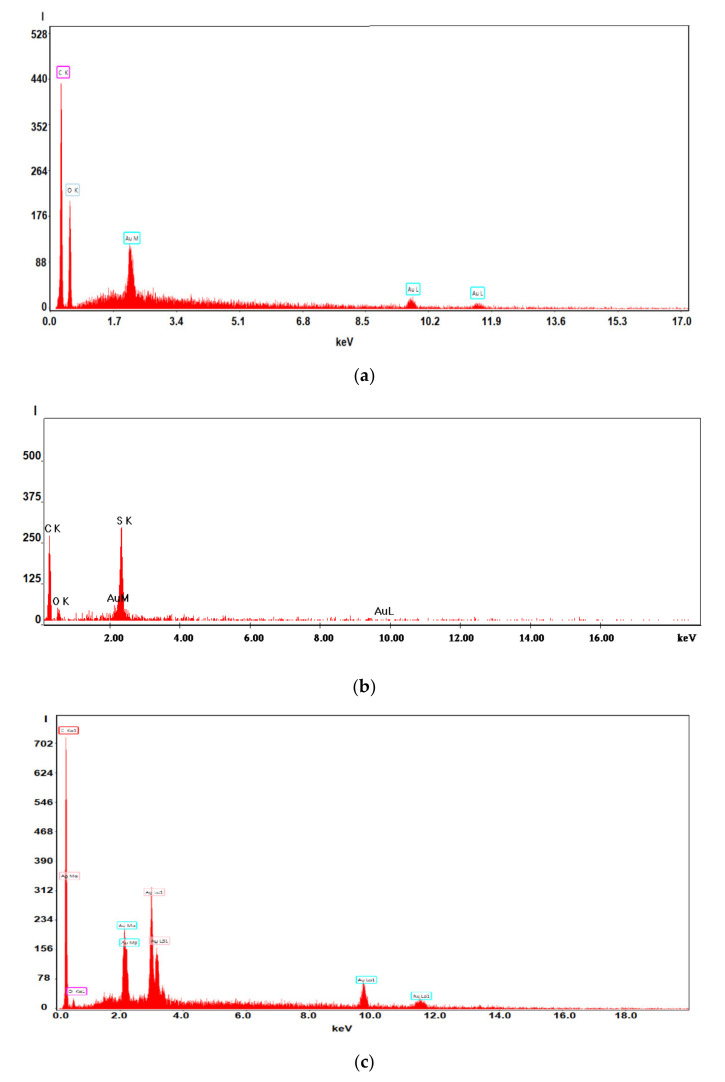
Energy Dispersive X-Ray Analysis (EADX) of the membranes on: (**a**) impregnated membranes; (**b**) processed impregnated membranes; (**c**) cross-section impregnated membranes.

The surface morphology and elemental composition of the membranes were analyzed using a scanning electron microscope (SEM; Figure 7) and energy dispersive X-ray analysis (EDX or EDAX). Additionally, the membrane samples were dried properly prior to the analysis and were coated with a sputtered gold layer of 400 Å (Figure 8).

### 3.2. Thermal Analysis

**Figure 9:**
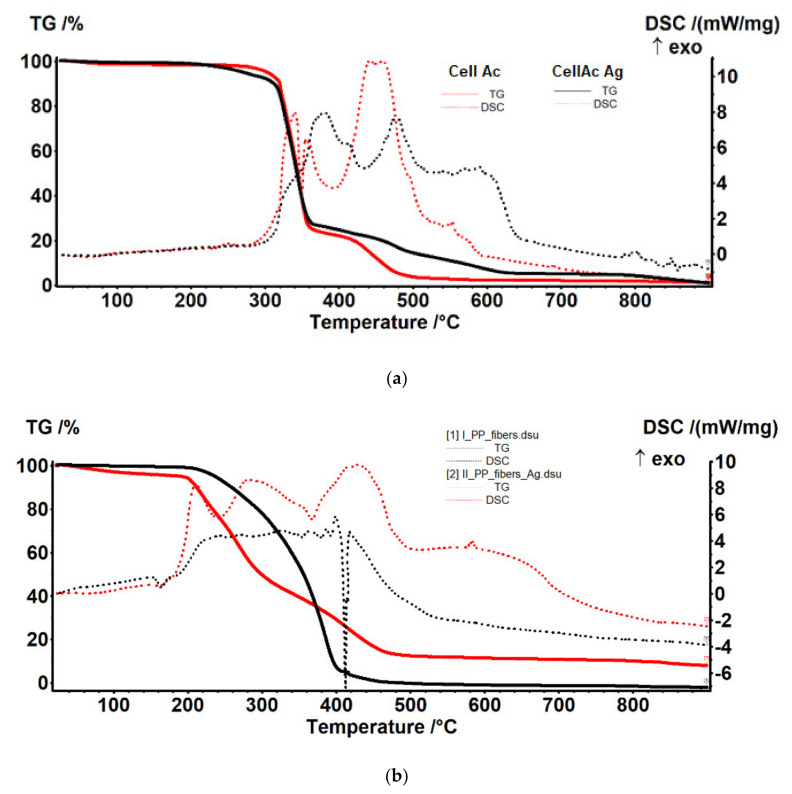
Thermal diagrams for: (**a**) cellulose acetate and Ag-cellulose acetate; (**b**) polypropylene fibers and impregnated polypropylene fibers.

The thermal analysis generated the results materialized in the diagrams presented in Figure 9, representing analysis results (TG and DSC) for cellulose acetate, Ag-cellulose acetate, polypropylene fibers, and impregnated propylene fibers. The diagrams were recorded up to 900 °C, with a heating speed of 10 °C/min.

### 3.3. FTIR Analysis

**Figure 10:**
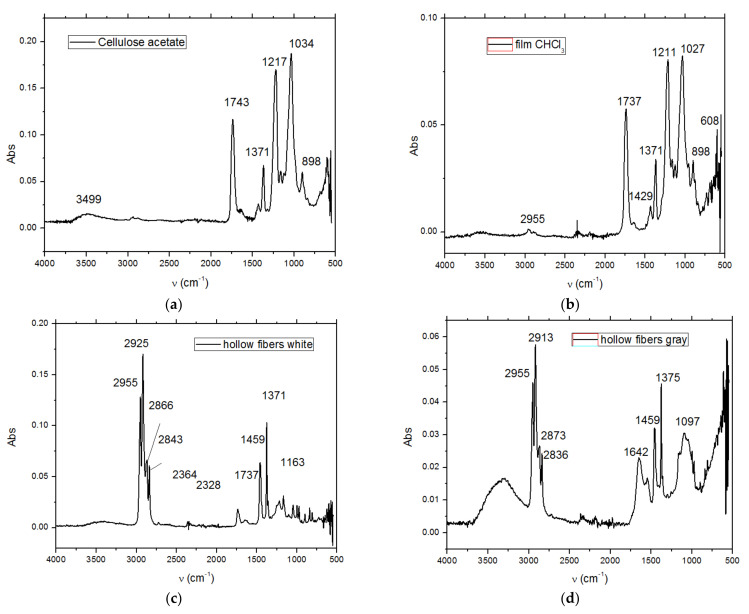
The Fourier-transform infrared spectrometry (FTIR) spectrum of (**a**) cellulose acetate; (**b**) Ag-cellulose acetate; (**c**) polypropylene fiber; and (**d**) impregnated polypropylene fiber.

Figure 10 presents the Fourier-transform infrared spectrometry (FTIR) spectra of the raw materials and obtained membranes (standard cellulose acetate, recovered cellulose acetate, polypropylene fibers, and polypropylene fibers impregnated with recovered cellulose acetate). The characterization was obtained directly from the solid samples by using the Bruker Tensor 27 with ATR diamond for the materials in this study.

### 3.4. Pollutant Removal Process Performance

**Figure 11:**
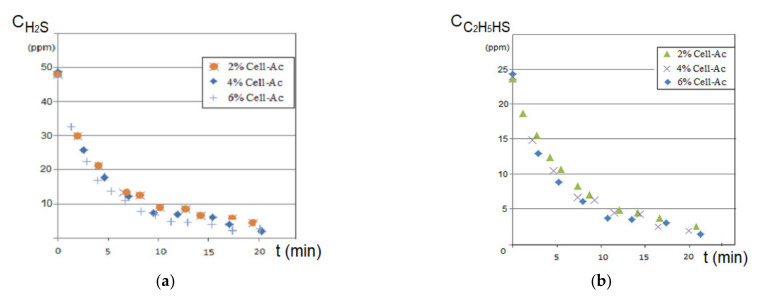
Variation of source phase concentration depending on the operating time at pH_SP_ = 5 and pH_RP_ = 12: (**a**) hydrogen sulfide and (**b**) ethanethiol.

**Figure 12:**
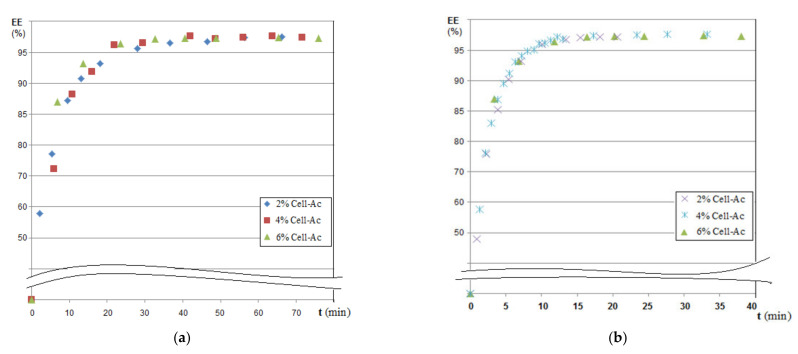
Dependency of extraction efficiency on time for various compositions of impregnated membranes at pH_SP_ = 5 and pH_RP_ = 12: (**a**) hydrogen sulfide and (**b**) ethanethiol.

**Figure 13:**
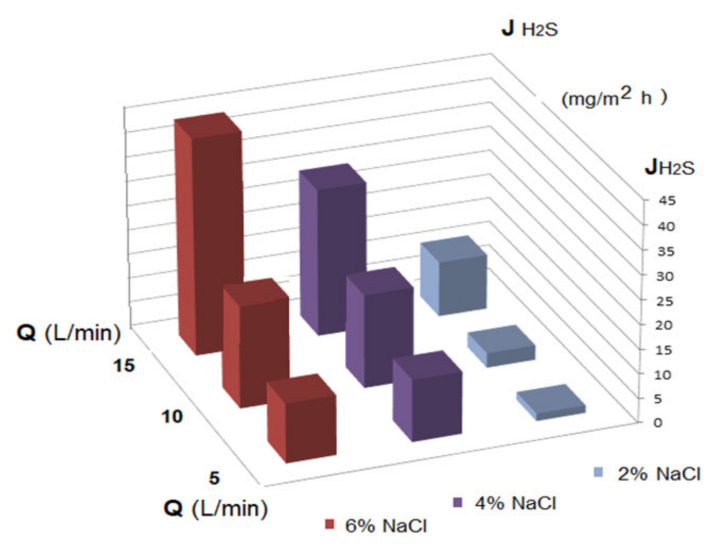
Dependency of the hydrogen sulfide flux variation on the electrolyte concentration (NaCl) in the source phase (at pH_SP_ = 5 and pH_RP_ = 12) for three recirculation rates (Q) of the source phase.

The results of hydrogen sulfide and ethanethiol permeation through the prepared membranes are given in Figure 11, Figure 12 and Figure 13. The main operating parameters and their influence on the evolution of the target chemistry species separation are presented.

## 4. Discussion

### 4.1. Membrane Materials Available for Study

#### 4.1.1. Polypropylene Support Membranes

The polypropylene microporous fibers considered in the present study were well known to the team, having been used in previous studies of biological treatment of municipal waters [83] or in the correction of the acidity and aluminum and copper content from the condensation of the individual thermal power plants of medium capacity (50–150 kW) [84].

**Figure 14:**
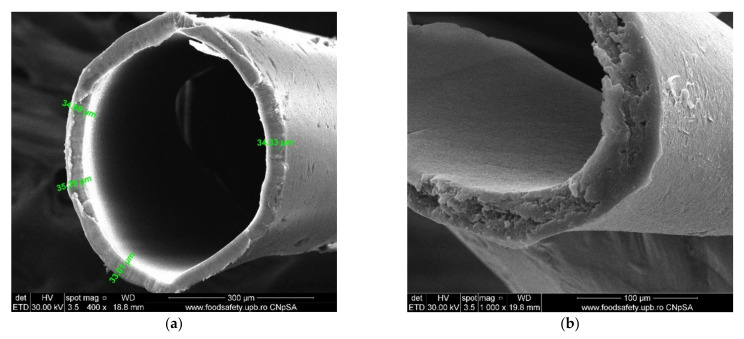
SEM images of the cross-section on polypropylene support fiber: (**a**) specific dimension; (**b**) a detail.

**Figure 15:**
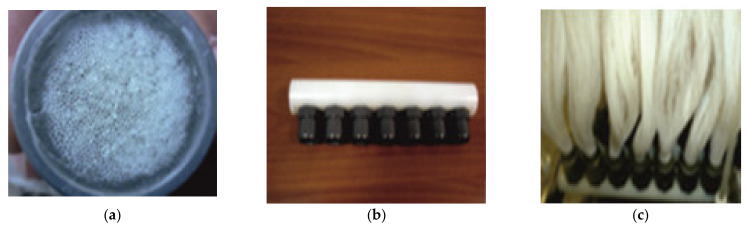
Assembly of polypropylene fibers: (**a**) encapsulation of the end of a beam; (**b**) linear beam interconnection support; (**c**) the beams in the linear support.

**Figure 16:**
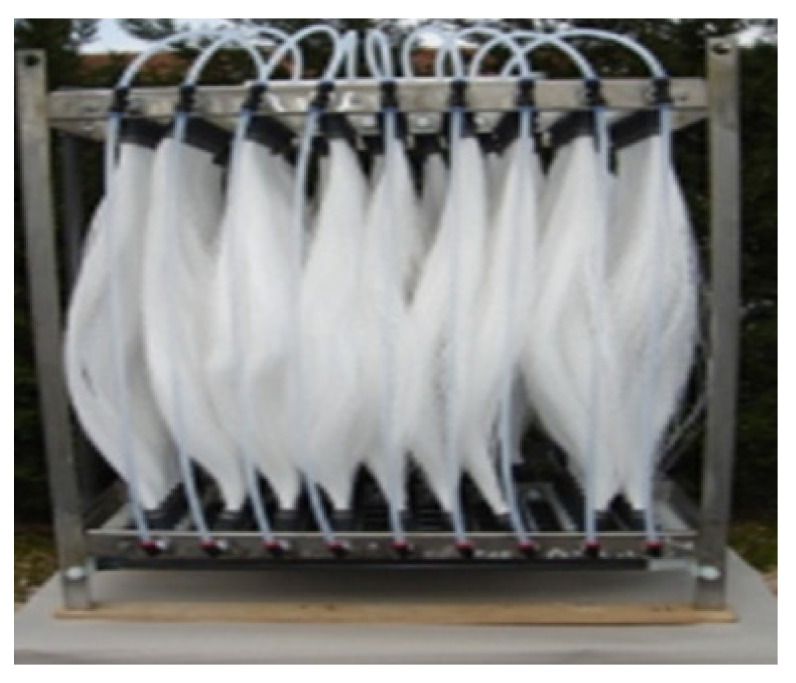
The permeation module (100 m^2^/m^3^).

The advantages of using this support material are:

- The complete characterization of microfiber and the large contact surface with effluents (1 m^2^/beam; Figure 7 and Figure 14);
- The possibility of scaling up the installation through a simple interconnection of the beams (Figure 15), which is technically unlimited (Figure 16).

Basically, a mass transfer surface of 1.0 m^2^/beam was easily achievable by using 100 fascicles, each having 100 m^2^ of active surface on 1.0 m^3^ of the permeation module. The installation presented in Figure 4 relates to a study performed upon a fiber bundle (of 1.0 m^2^).

This module worked for 30 months in a teaching unit (between 2015 and 2017), serving the analytical chemistry and inorganic qualitative analysis laboratories as well as two research laboratories in the field of membranes and membrane processes (liquid membranes and preparation of polymeric membranes from solutions): low alkaline solution, fresh air.

#### 4.1.2. Used Photographic and Cinematographic Films

The photographic material considered in the study was used in the art of photography and cinematography in the form of rigid transparent support or photographic plate (known as roll film), this being the actual flexible transparent support. If used for a long time, the cellulose acetate support (now abandoned) has the quality of not being flammable and has a long aging period. In the end, though, by aging or mishandling it becomes stiff, crusty, and brittle [85].

In Romania, cinematographic/photographic films based on cellulose acetate were used for a long time, especially during the Ceaușescu period when tens of thousands of propagandistic movies and pictures were made. After 1990, because of both obsolescence and physical-chemical degradation, important film warehouses were instituted, of approximate 200–250 tons, which are still available today. The films used in that period can be classified according to several criteria: geometric dimensions (see next paragraph); purpose (negative and positive films, counter type, or sound films, etc.); spectral sensitivity (un-sensitized, orthochromatic, panchromatic, infra-chromatic, and color) [86,87].

Given the dimensions, there are films of 70 mm/65 mm, 35 mm, 32 mm (2 × 16 mm^2^), 16 mm, and 8 mm. The 70 mm films were used in widescreen systems in two variants: the negative and positive are 70 mm, or the negative is 65 mm and the positive is 70 mm. By 1980–1990, 70 mm films were used less and less, the less expensive version of 35 mm films being preferred. Nevertheless, the advantages of large format film remain and are mainly given by the higher resolution [87]. 

The characterization by electronic microscopy of representative samples: veiled films, exposed or wrongly conditioned films, or used films showed that, in addition to the cellulose acetate support material, dispersed, agglomerated, or even evenly distributed silver nanoparticles were observed (Figure 6a–j). Thus, the uniform distribution of silver nanoparticles was more obvious in veiled films (totally exposed to light, Figure 6a,b), along with the relatively small amount of silver compared to that of acetate film. The same degraded film presents multiple micro-cracks of 10–15 nm wide and 1–5 µm long (Figure 6i), and the measured size of the silver nanoparticles is 20–35 nm (Figure 6j. Various degraded film samples (Figure 6c–g) present various distributions and agglomerations of silver nanoparticles of 15–45 nm (Figure 6d,f,h) as well as nanometric micro-cracks with measurable but irregular micrometric lengths. These micro-cracks cause the films to be brittle, difficult to handle and unroll. On the other side, the micro-cracks favor the grinding process of the samples at the colloidal mill with ceramic balls, shortening the grinding time from three hours for non-degraded (young) films to at most one hour for aged films. 

The composition of the used films taken into consideration was determined using Fourier-transform infrared spectrometry (FTIR) comparing the spectrum of standard cellulose acetate (Figure 10a) with that of a cellulose acetate film containing silver (the raw material, Figure 10b), while the thermal behavior was determined by thermo-gravimetry (TG) and differential thermal analysis (DSC; Figure 9a). From the comparative analyses, FTIR, TG, and DSC, the composition in cellulose acetate, and the different behavior of the films containing silver nanoparticles (Figure 6 correlated with Figure 9a and Figure 10a,b) were confirmed by the slight displacement of the absorption maxima (FTIR) and the appearance of additional thermal effects (DSC).

**Figure 17:**
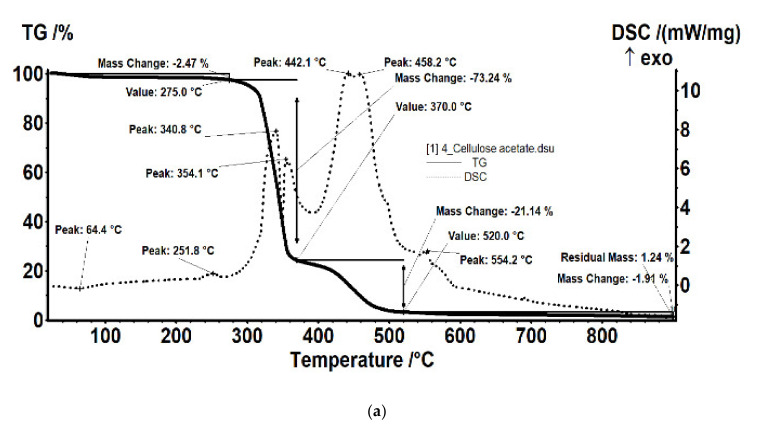

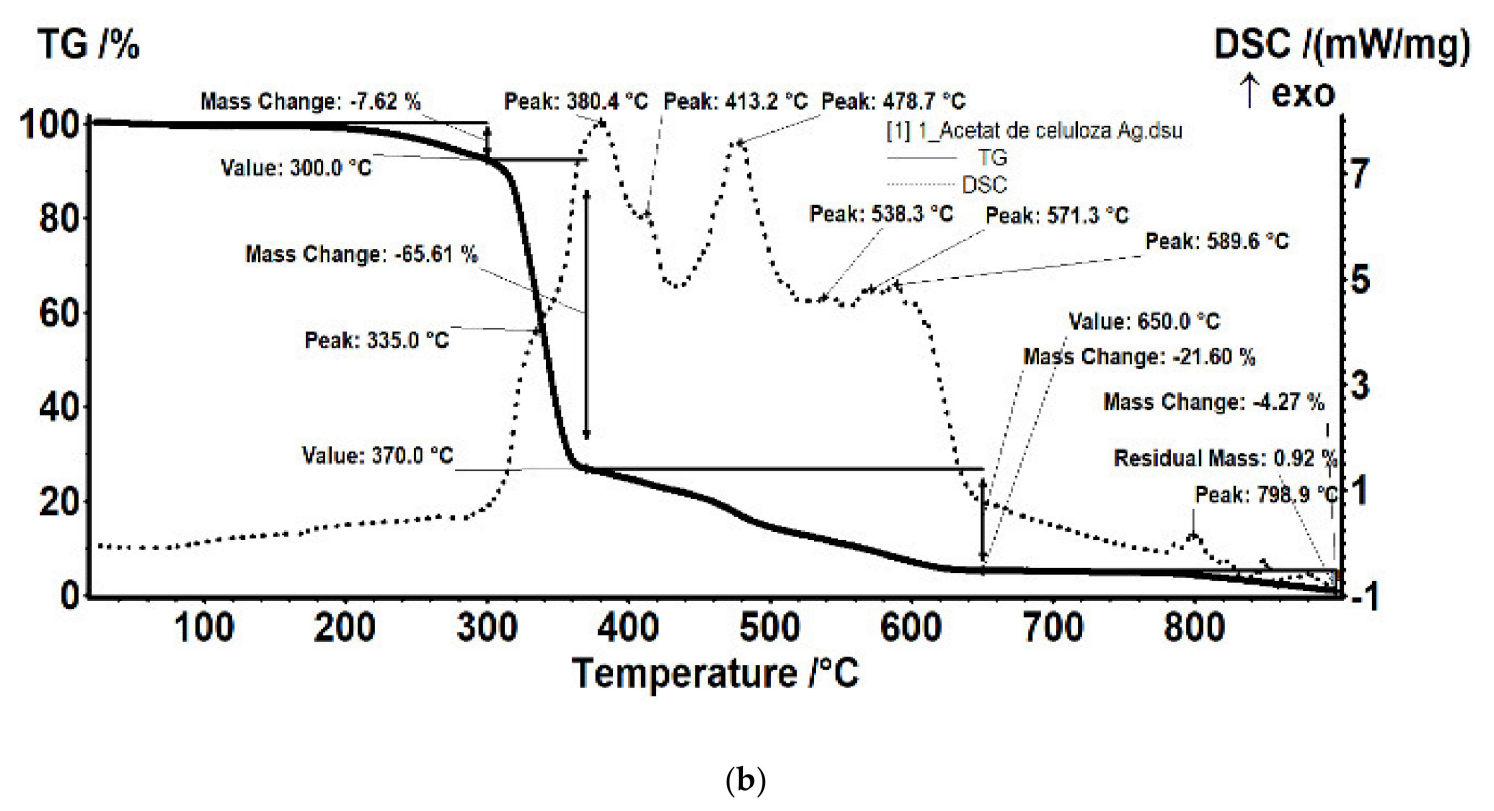
Details of the thermal analysis of the samples of cellulose acetate (**a**) and cellulose acetate with silver nanoparticles (**b**).

For the film sample containing silver, it was interesting to detail the thermal study compared to the support film (without silver nanoparticles; Figure 17).

The sample (Figure 17a) was thermally stable up to 275 °C. The sample lost 2.47% of its initial weight, most likely water (moisture) absorbed, the process wasaccompanied by a weak endothermic effect at 64.4 °C. A weak exothermic effect was also observed at 251.8 °C, corresponding to a process of partial oxidation of the organic molecule. Degradative oxidation began at 275 °C, the sample losing 73.24% of its initial weight until the temperature reached 370 °C. The process was accompanied by two separate exothermic peaks, at 340.8 °C and 354.1 °C, corresponding to the degradation of the acetate and cellulose group. The carbon mass remaining after initial degradation was eliminated in the range of 370–520 °C by oxidation, the process being accompanied by an intense, broad exothermic effect with two maxima at 442.1 °C and 458.2 °C.

The sample of cellulose acetate with silver nanoparticles (Figure 17b) began to lose weight after 150 °C, by 300 °C it had lost 7.62% of its initial weight through an oxidative process. The process was accompanied by a weak exothermic effect with a maximum of 269.1 °C. The main degradation process took place between 300 and 370 °C, when 65.61% of the initial mass was lost. The weight loss continued slower after 370 °C, up to 650 °C when 21.60% of the initial mass was removed. The oxidative degradation processes were accompanied by intense, broad, overlapping exothermic effects, with maxima at 335, 380.4, 413.2, 478.7, 538.3, 571.3, and 589.6 °C. The first effects corresponded to the rapid oxidation of organic matter and, towards the end of the interval, the burning of the carbonic residue took place.

The idea of recovering individual silver or cellulose acetate from these used films proved to be not only technically ineffective but also polluting due to physical-chemical processes by which the polymer was recovered or by which the silver was solubilized.

In this paper, the study of the integral use of the film was initiated, both acetate and silver, by solubilizing at concentrations of 2%, 4%, and 6% of the film, in methylene chloride: methanol mixture using a 2:1 volumetric ratio. Concentrations above 6% of cellulose acetate in this solvent mixture became difficult to use in the process of impregnating propylene support fibers, which were carried out by capillary adsorption. Although in the laboratory workshops a mixture of methylene chloride and methanol was used, mainly because of speed and simplicity in handling, cellulose acetate could be recovered on a pilot scale and with environmentally friendly solvents [72].

#### 4.1.3. Membrane Characterization

Figure 7 and Figure 8 present the results of the SEM, HF SEM, and EDAX studies for polypropylene support fiber (PPMS) and Ag-cell acetate composite membrane on polypropylene support (Ag-Cell-Ac-PPM) both before and after retention of target substances. For the PPMS support membrane at two resolutions, submicron pores well-distributed on the surface were highlighted (Figure 7a,b). This favors the adsorption of the polymeric solution obtained from photo films. For the Ag-Cell-Ac-PPM composite membrane, SEM and HF SEM images showed that the pores were coated (filled) with cellulose acetate (Figure 7c,d), and EDAX analysis confirmed the composition (Figure 8). It is interesting that, although the silver presence in the polymeric solution was obvious considering both the source films (Figure 6b,d,f) and the appearance of the fibers (Figure 7d,f), it did not appear in the EDAX spectrum (Figure 8a,b) being most likely masked by the gold coating from the preparation of the samples for SEM examination. Regarding the composite membranes Ag-Cell-Ac-PPM which were used, the images from the scanning electron microscopy revealed the surface contamination (the appearance of some agglomerations) but also the presence of sulfur in the EDAX spectrum, which indicated adsorption of the target substances both in-depth and on the surface (Figure 8b).

It is a very interesting fact that, in the hollow fiber section (Figure 7g,h), the presence of silver nanoparticles could be evidenced by EDAX (Figure 8c).

The amount of cellulose acetate in the polypropylene fibers increased with increasing concentration of the solution in the film (Table 4) but the increase was a negative deviation from linearity, indicating that the adsorption of concentrated solutions was more difficult than the 2% solution. This practical aspect was important both for the preparation of the composite membrane, but also for the transition to the pilot-scale when the change of the solubilizing solvent of the films must avoid the increase of the viscosity of the cellulose acetate solution.

**Figure 18:**
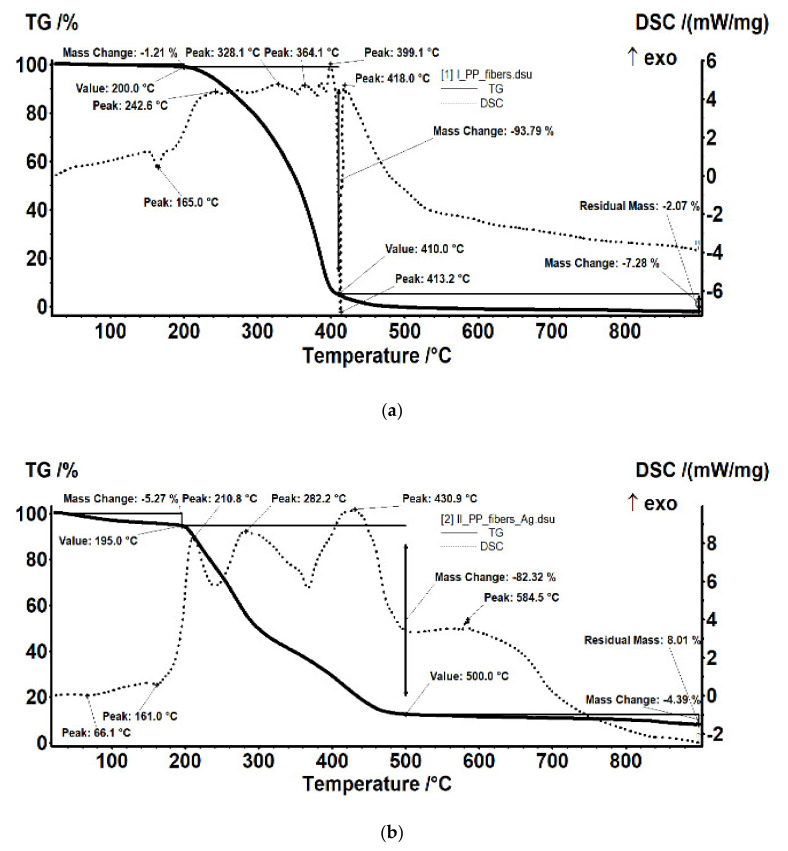
Details of the thermal analysis (thermo-gravimetry (TG) and differential thermal analysis (DSC)) of (**a**) the support fibers; (**b**) the support fibers impregnated with cellulose acetate containing silver nanoparticles.

The comparative FTIR analysis of polypropylene fibers and impregnated polypropylene fibers (Figure 10c,d) clearly showed the presence of cellulose acetate in the pores of the impregnated fiber. At the same time, the thermal diagrams of the membranes impregnated on the polypropylene fibers were also suggestive and interesting, indicating the thermal field of use (Figure 18) as follows:

- The polypropylene support test (Figure 18a) was stable up to 200 °C. At 165 °C, an endothermic effect was recorded without weight loss, which corresponded to the melting of PP. After 200 °C the process of oxidative degradation took place, the weight loss recorded up to 410 °C being 93.79%. The process was accompanied by a series of superimposed exothermic effects, with peaks at 328, 364, or 399.1 °C. After the degradative oxidation, the carbon residue was burned during an exothermic process with a maximum value of 418 °C.
- The fibers impregnated with silver (Figure 18b) suffered a weight loss of 5.27% up to 195 °C (probably due to a precursor with which they were impregnated). The melting point was only 161 °C, lower than that of the impregnated fibers. The oxidative degradation started at 195 °C so that at 500 °C the sample lost 82.32% of its weight. The process was accompanied by three broad, intense, and partially overlapping exothermic effects, with peaks at 210.8, 282.2, and 430.9 °C. The carbon residue was burned after 500 °C, the process being accompanied by a wide exothermic effect, with a maximum value of 584.5 °C.

If the use of the cellulose acetate was usually allowed for temperatures up to 250 °C then, once introduced into the pores of the polypropylene membrane, the temperature up to which it could be used decreased by 100 °C, accentuated by the presence of silver nanoparticles, which activated the decomposition.

### 4.2. Removal of Foul-Smelling Pollutants 

#### 4.2.1. Removal of Hydrogen Sulfide and Ethanethiol from Synthetic Source Phases 

The experiments performed for the removal of hydrogen sulfide and ethanethiol from synthetic source phases started from the results reported in specialty literature regarding the influence of the pH of the source and receptor phases on the membrane system [70,71,72,73], thus establishing a pH = 5 for the source phase and a pH = 12 for the receiving phase.

Simultaneously, an initial hydrogen sulfide or ethanethiol concentration was considered in the source phase starting at the sodium sulfide stock solution of 150 mg/L or from the ethanethiol stock solution of 50 mg/L. Thus, through dilution and in-situ pH adjustments, solutions of desired concentrations could be obtained for the source phase. For the present case: 50 ppm H_2_S and 25 ppm ethanethiol, which was verified both spectrophotometrically and with specialized sensors.

The volume of the source phase can be chosen between 12 and 50 L according to the experiment, while the volume of the receiving phase, 0.01 mol/L NaOH solution, was in all cases set to 1.0 L. 

In the case of hydrogen sulfide solution or sodium hydroxide receiving solution, as well as in the case of ethanethiol retention experiments, the recirculation flow of the source phase could be adjusted, with the volumetric pump, between 1 and 20 L/min.

The flow rate of the receiving phase in the case of hydrogen sulfide removal experiments was 0.1–0.2 L/min.

#### 4.2.2. The Influence of the Initial Cellulose Acetate Concentration

To determine the influence of the initial concentration of the cellulose acetate solution used in the preparation of Ag-cellulose composite membranes of polypropylene support on the removal of hydrogen sulfide from the 50 ppm concentration solution, a flow rate of a recirculation of 1.0 L/min and initial pH of 5 were set. The receiving phase was recirculated using a flow rate of 0.10 L/min for an initial pH of 12. The operation took place in a laboratory installation (Figure 5) using an operating scheme having the source phase outside the capillaries.

The hydrogen sulfide concentration decreased drastically in the first 5 to 7 min of the operation, after which the decrease was flattened suggesting a decrease in the hydrogen sulfide flow through the membrane, determined by the decrease of the concentration gradient and by saturating the membrane with hydrogen sulfide (Figure 11a). The higher cellulose acetate concentration in the composite membrane favored the removal of the hydrogen sulfide, being more obvious in the first 7 to 10 min (Figure 11a correlated with Table 4).

To remove the ethanethiol from the entrained air, the installation presented in Figure 5 was used. It also operated based on the schema that used the receiving phase inside the fibers at a debit of 0.10 L/min and a debit of 10 L/min source phase (outside the fibers).

The results indicated, in the first 10 min, a pronounced decrease of the thiol content, followed by the flattening of the concentration value for the next interval (Figure 11b). The concentration of the cellulose acetate solution used in the preparation of the composite Ag-acetate membranes of polypropylene support (Ag-Cell-Ac-PPM) on the removal of thiol had a similar dependency on the removal of hydrogen sulfide in the first 5 to 7 min but was more attenuated than in the previous case suggesting that the mass transfer was imposed by the extraction in the alkaline receiving phase (Figure 11b correlated with Table 4).

The prolonged operation of the considered systems was reflected in the dependencies of the process efficiency (Figure 12a,b and Table 5).

Processing of polluted systems with foul-smelling gases with Ag-cellulose acetate composite capillary membranes on the polypropylene support (Ag-Cell-Ac-PPM) must be done for a maximum of 20 min, after which it became ineffective. This suggested either the transfer of the depleted source phase to another membrane module (beam) or the refresh of the source and/or receiving phases to maintain the concentration gradient, which obviously governs the process.

#### 4.2.3. Influence of Recirculation Flow and Electrolyte Concentration (NaCl) for the Source Phase

The hydrodynamic regime of the phases through the membrane fibers was relatively narrowed by the fact that it was governed by the phenomena of flow through the capillaries, but the flow of the phases outside the membrane fibers could cover a wide range from laminar to turbulent to obtain maximum extraction efficiency (removal) of the pollutant generating the unpleasant odor.

The performed experiments showed that the flow of hydrogen sulfide through the membrane increased both with the increase of the recirculation flow and of the inert electrolyte concentration (Figure 13).

The optimal value of the hydrogen sulfide flow was 40 mg H_2_S/m^2^ h, under the conditions imposed by the pH of the source and receiving phases, as well as by using the most favorable membranes from this study.

This optimal value was obtained when the following conditions were achieved:

- pH of the source phase: 5;
- pH of the receiving phase: 12;
- initial hydrogen sulfide concentration: 50 ppm;
- electrolyte concentration (NaCl): 6%;
- the initial concentration of the cellulose acetate solution: 6% (for Ag-Cell-Ac-PPM);
- the recirculation flow of the source phase: 15 L/min;
- receiving phase flow: 0.25 L/min.

#### 4.2.4. Initial Tests for Retaining Odor—Generating Pollutants at Pilot Level

**Figure 19:**
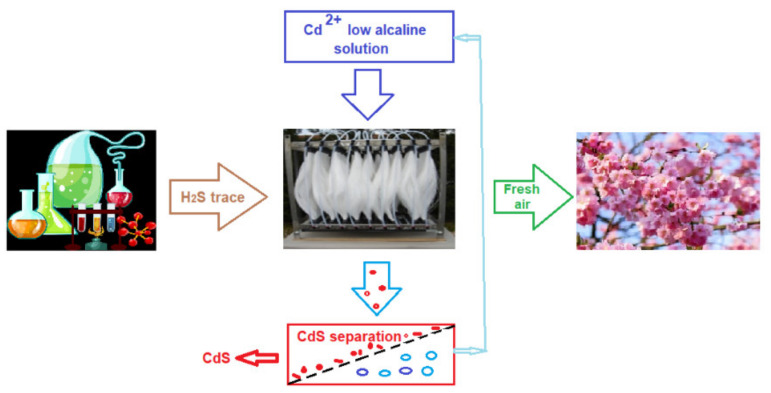
Schematic presentation of the initial test for odor control with the permeation module (100 m^2^/m^3^).

During 2015–2017, for 30 months, a pertraction module of 100 m^2^/m^3^ (Figure 16) was used in the suggestive exhaust scheme installation (Figure 19) of three analytical chemistry laboratories: qualitative inorganic, analytical chemistry (having a surface of 2 × 30 m^2^), and research in the field of membranes (with a surface of 80 m^2^), all having a height of 6 m. Following the test, 1234 g of cadmium sulfide (CdS) was obtained. 

The module managed to keep the concentration of hydrogen sulfide eliminated in the atmosphere below the limits of un-pleasant odor (0.13 ppm). Unfortunately, in Romania, the accepted limit for the hydrogen sulfide in air is 15 mg/m^3^ [88].

## 5. Conclusions

The bad smell of the air in metropolitan areas is generated by multiple sources of industrial-economic agents, including those dealing with the storage, treatment, and recycling of various wastes. The special situation of the Bucharest metropolitan area caught attention through numerous deviations from air quality, including unbearable odor. 

This paper presented aspects regarding the possibility of correcting the air odor using cellulose acetate membranes impregnated with silver in polypropylene fibers. The cellulose acetate used was waste from the film industry (contains silver nanoparticles). 

The impregnated membranes obtained were morphologically and structurally characterized (SEM, HF SEM, EDAX, FTIR, TG, ATD) but also in terms of performance in the process of retaining hydrogen sulfide and ethanethiol (as foul-smelling gas-generating target substances). The membrane process must be used to remove the target substances at the source (synthetic solutions were used) because after dispersion in the air, the removal was difficult and both technically and economically inefficient.

The solution chosen for the design of the membrane module was based on impregnated fiber bundles having a mass transfer surface of 1 m^2^/bundle and 100 m^2^/m^3^ when scaled up (100 bundles per pertraction unit).

All obtained results showed that the target substances could be removed with an efficiency of over 95% from synthetic solutions of 50 ppm H_2_S or 25 ppm C_2_H_5_SH. The flow of chemical species through membranes depended on the amount of cellulose acetate impregnated in the polypropylene fibers, the flow regime of the source phase, and the concentration of electrolytes (NaCl).

## Figures and Tables

**Table 1:** Source of the different gaseous chemicals that produce unpleasant odors.

Source	Gaseous Components						
	H_2_S	Mercaptans	NH_3_	CH_4_	SO_2_	Phenols	Others
Biogas: CH_4_, H_2_S, mercaptans	+	+	−	+	n/a	−	−
Pulp and paper: CH_3_SH; (CH_3_)_2_S; SO_2_	+	+	−	n/a	+	−	−
Nitrogen-Phosphate based fertilizers: NH_3_; SO_2_; F_2_	−	−	+	−	+	−	F_2_
Pesticides: CH_3_CHO; NH_3_; H_2_S; phenols	+	−	+	−	−	+	CH_3_CHO
Raw hides and skins storage: NH_3_; H_2_S	+	−	+	−	−	−	n/a
Finishing operations: H_2_S; CH_4_	+	−	−	+	−	−	−
Sugar and distillery bio-methanation: H_2_S; NH_3_	+	−	+	−	−	−	−
Chemical: NH_3_; H_2_S; Cl_2_; mercaptans; phenols	+	+	+	−	−	+	Cl_2_
Dye and dye intermediates: NH_3_; H_2_S; SO_2_; mercaptans	+	+	+	n/a	+	−	−
Bulk drugs, pharmaceuticals biological extracts: H_2_S, SO_2_, mercaptans	+	+	−	−	+	+	−
Wastewater treatment plant anaerobic decomposition: H_2_S; mercaptans	+	+	−	−	−	−	−
Waste resulting from plant distillation	+	+	+	n/a	n/a	+	
Municipal solid waste anaerobic decomposition: H_2_S, mercaptans	+	+	−	+	−	−	−
Waste storage effluent treatment plant: CH_4_, H_2_S, mercaptans	+	+	−	+	−	−	−

n/a: not applicable.

**Table 2:** The removal, separation, or recovery of H_2_S by membrane processes.

Material Membranes	Feed System	H_2_S	Refs.
Polymers	Solution/gases	removal	[34,53,54,55,56]
Metal organic framework	gases	removal	[57]
Salt hydrate chemical absorbents	gas streams	separation	[58]
Functionalized carbon nanotubes	gases	separation	[59]
Zeolites as a filler	gases	separation	[60]
Vegetable oil-polyurethane	gases	separation	[61]
Polymeric contactors	gases	removal	[62,63]
Cobalt oxide silica	gases	separation	[64]
Polydimethylsiloxane	biogas	removal	[65]
Hybrid membrane	biogas	removal	[66]
Various adsorbents	gases	capture	[67]
Imidazolium ionic liquids	acid gases	removal	[68]
Lipid	gases	permeation	[69]
Hollow fiber contactors	gases	removal	[70]
Porous Organic Polymer	natural gas	selective removal	[71]

**Table 3:** Main features of the membranes (GOST Ltd.).

Material	Porosity (%)	Dimension of Pore (µm)	External Diameter (mm)	Fascicle Dimensions (mm)	Filtration Surface (Fascicle) (m^2^)	pH	T_max_ (°C)
Polypropylene (PP)	40–50	0.002–0.2	0.45	25 × 750	1.0	1–14	50

**Table 4:** The amount of cellulose acetate in a bundle of composite membranes depending on the concentration of the fiber impregnation solution.

Cellulose Acetate Solution Concentration (%)	2	4	6
The amount of cellulose acetate in a bundle of composite membranes (g)	8.41 + 0.22	15.16 + 0.34	23.63 + 0.45

**Table 5:** Efficiency extraction of hydrogen sulfide or ethanethiol on repeatable experiments.

Membrane with Cell Ac Ag. (Ag-Cell-Ac-PPM)	Efficiency Extraction (%)	
	Hydrogen Sulfide	Ethanethiol
2%	89 ± 3	91 ± 2
4%	91 ± 3	92 ± 2
6%	94 ± 3	95 ± 2

## References

[1] Katz M. (1955). Atmospheric pollution: A growing problem in public health. AJPH.

[2] Beck J.F., Cormier F., Donini J.C. (1979). The combined toxicity of ethanol and hydrogen sulfide. Toxicol. Lett..

[3] Layton D.W., Cederwall R.T. (1986). Assessing and managing the risks of accidental releases of hazardous gas: A case study of natural gas wells contaminated with hydrogen sulfide. Environ. Int..

[4] Alfonsin C., Lebrero R., Estrada J.M., Munoz R., Kraakman N.J.R., Feijo G., Moreira M.T. (2015). Selection of odour removal technologies in wastewater treatment plants: A guideline based on life cycle assessment. Environ. Manag..

[5] Manisalidis I., Stavropoulou E., Stavropoulos A., Bezirtzoglou E. (2020). Environmental and Health Impacts of Air Pollution: A Review. Front. Public Health.

[6] Prada M., Prada I.F., Cristea M., Popescu D., Bungau C., Aleya L., Bungău C.C. (2020). New solutions to reduce greenhouse gas emissions through energy efficiency of buildings of special importance—Hospitals. Sci. Total Environ..

[7] Bungau C.C., Prada I.F., Prada M., Bungau C. (2019). Design and operation of construction: A healthy living environment-parametric studies and new solutions. Sustainability.

[8] Li F., Liu Y., Lü J., Liang L., Harmer P. (2015). Ambient air pollution in China poses a multifaceted health threat to outdoor physical activity. J. Epidemiol. Community Health.

[9] Portney P.R. (1990). Air pollution policy. Public Policies for Environmental Protection.

[10] Robinson E., Robbins R.C. (1970). Gaseous sulfur pollutants from urban and natural sources. J. Air Pollut. Control Assoc..

[11] Schnelle K.B., Dunn R.F., Ternes M.E. (2016). Pollutant Removal Processes. Air Pollution Control Technology Handbook.

[12] Edelman S. (1966). The Law of Federal Air Pollution Control. J. Air Pollut. Control Assoc..

[13] Schiffman S.S., Williams C.M. (2005). Science of Odor as a Potential Health Issue. J. Environ. Qual..

[14] Bergen J.V. (1958). Industrial Odor Control. J. Air Pollut. Control Assoc..

[15] Ryltseva Y., Orlov V. (2020). Measures to prevent sewerage odor emissions into the atmosphere. IOP Conf. Ser. Mater. Sci. Eng..

[16] Borisenko A.V., Gazaliev A.M. (2006). Method for cleaning industrial gases from harmful impurities in a high-intensity electric field. J. Chem. Chem. Technol..

[17] Midha V., Dey A. (2008). Biological treatment of tannery wastewater for sulfide removal. Int. J. Chem. Sci..

[18] Liu Y., Wu C., Zhou X., Zhu D.Z., Shi H. (2015). Sulfide elimination by intermittent nitrate dosing in sewer sediments. J. Environ. Sci..

[19] Li J., Ma X., Qu G., He K. (2019). Efficient purification of hydrogen sulfide by synergistic effects of electrochemical and liquid phase catalysis. J. Sep. Pur..

[20] Alcheikhhamdon Y., Hoorfar M. (2017). Natural gas purification from acid gases using membranes: A review of the history, features, techno-commercial challenges, and process intensification of commercial membranes. J. Chem. Eng. Process. Process Intensif..

[21] Wysocka I., Gębicki J., Namieśnik J. (2019). Technologies for deodorization of malodorous gases. Environ. Sci. Pollut. R..

[22] Kesting R. (1988). Synthetic polymeric membranes. J. Colloid Interface Sci..

[23] Baker W. (2012). Membrane Technology and Applications.

[24] Mulder M. (1996). Basic Principles of Membrane Technology.

[25] Kammermeyer K. (1976). Technical Gas Permeation Processes. Chem. Ing. Tech..

[26] Baker R.W. (2012). Membrane Transport Theory. Membrane Technology and Applications.

[27] Stannett V.T., Koros W.J., Paul D.R., Lonsdale H.K., Baker R.W. (1979). Recent advances in membrane science and technology. Chemistry.

[28] Guizard C., Rios G. (1996). Chapter 12 Transport and fouling phenomena in liquid phase separation with inorganic and hybrid membranes. Membr. Sci. Technol..

[29] Merkel T.C., Freeman B.D., Spontak R.J., He Z., Pinnau I., Meakin P., Hill A.J. (2002). Ultrapermeable, reverse-selective nanocomposite membranes. Science.

[30] Bazhenov S.D., Bildyukevich A.V., Volkov A.V. (2018). Gas-liquid hollowfiber membrane contactors for different applications. Fibers.

[31] Drioli E., Stankiewicz A.I., Macedonio F. (2011). Membrane engineering in process intensification—An overview. J. Membr. Sci..

[32] Iulianelli A., Drioli E. (2020). Membrane engineering: Latest advancements in gas separation and pre-treatment processes, petrochemical industry and refinery, and future perspectives in emerging applications. Fuel Process. Technol..

[33] Mulder M., Crespo J.G., Böddeker K.W. (1994). The Use of Membrane Processes in Environmental Problems. An Introduction. Membrane Processes in Separation and Purification.

[34] Silva A.F.R., Ricci B.C., Koch K., Weißbach M., Amaral M.C.S. (2020). Dissolved hydrogen sulfide removal from anaerobic bioreactor permeate by modified direct contact membrane distillation. Sep. Pur. Technol..

[35] Dardor D., Minier-Matar J., Janson A., AlShamari E., Adham S. (2020). The effect of Hydrogen sulfide oxidation with ultraviolet light and aeration on sour water treatment via membrane contactors. Sep. Pur. Technol..

[36] Gabelman A., Hwang S.T. (1999). Hollowfiber membrane contactors. J. Membr. Sci..

[37] Tabe-Mohammadi A. (1999). A Review of the Applications of Membrane Separation Technology in Natural Gas Treatment. Sep. Sci. Technol..

[38] Rappert S., Muller R. (2005). Odor compounds in waste gas emissions from agricultural operations and food industries. Waste Manag..

[39] Krayzelova L., Bartacek J., Díaz I., Jeison D., Volcke E.I.P., Jenicek P. (2015). Microaeration for hydrogen sulfide removal during anaerobic treatment: A review. Rev. Environ. Sci. Biotechnol..

[40] Samimi A., Zarinabadi S., Bozorgian A., Amosoltani A., Esfahani M.S.T., Kavousi M.K. (2020). Advances of Membrane Technology in Acid Gas Removal in Industries. Prog. Chem. Biochem. Res..

[41] Pochwat K., Kida M., Ziembowicz S., Koszelnik P. (2019). Odours in Sewerage—A Description of Emissions and of Technical Abatement Measures. Environments.

[42] Fang J.J. (2012). Odor compounds from different sources of landfill: Characterization and source identification. Waste Manag..

[43] Aatamila M., Verkasalo P.K., Korhonen M.J., Suominen A.L., Hirvonen M.R., Viluksela M.K., Nevalainen A. (2011). Odour annoyance and physical symptoms among residents living near waste treatment centers. Environ. Res..

[44] Zhang L., Sedlak L.D. (2012). A framework for identifying characteristic odor compounds in municipal wastewater effluent. Water Res..

[45] Hancock J.T. (2019). Hydrogen sulfide and environmental stresses. Environ. Exp. Bot..

[46] Antonelli D., Sabanchiev A., Rosner E., Turgeman Y. (2014). Sewer gas induced myocardial toxicity. Harefuah.

[47] Samuel A.D., Bungau S., Tit D.M., Melinte C.E., Purza L., Badea G.E. (2018). Effects of long term application of organic and mineral fertilizers on soil enzymes. Rev. Chim..

[48] Ginghina R.E., Bojin D., Tiganescu T.V., Petrea N., Bungau S., Mosteanu D.-E. (2020). Research on the efficiency of testing a new adsorbent material with cellulose structure for the depollution of waste water. Mater. Plast..

[49] Moisa C., Copolovici L., Bungau S., Pop G., Imbrea I., Lupitu A., Nemeth S., Copolovici D. (2018). Wastes resulting from aromatic plants distillation—Bio-sources of antioxidants and phenolic compounds with biological active principles. Farmacia.

[50] https://www.mediafax.ro/social/sarbatoare-cu-poluare-nordul-capitalei-cel-mai-irespirabil-loc-de-pe-planeta-indicele-nivelului-de-poluare-in-noaptea-de-craciun-19820504.

[51] https://www.digi24.ro/stiri/sci-tech/natura-si-mediu/date-alarmante-poluarea-din-nordul-capitalei-a-atins-cote-mai-mari-decat-in-bangladesh-cea-mai-poluata-tara-din-lume-1423834.

[52] Szałata Ł. (2020). Impact of Implementing a Deodorization System on the Functioning of a Waste Management Plant. Sustainability.

[53] Wiheeb A.D., Shamsudin I.K., Ahmad M.A., Murat M.N., Kim J., Othman M.R. (2013). Present technologies for hydrogen sulfide removal from gaseous mixtures. Rev. Chem. Eng..

[54] Bernardoa P., Drioli E. (2010). Membrane Gas Separation Progresses for Process Intensification Strategy in the Petrochemical Industry. Pet. Chem..

[55] Alqaheem Y., Alomair A., Vinoba M., Pérez A. (2017). Polymeric Gas-Separation Membranes for Petroleum Refining. Int. J. Polym. Sci..

[56] Adams R., Carson C., Ward J., Tannenbaum R., Koros W. (2010). Metal organic framework mixed matrix membranes for gas separations. Micropor. Mesopor. Mater..

[57] Quinn R., Appleby J.B., Pez G.P. (2002). Hydrogen sulfide separation from gas streams using salt hydrate chemical absorbents and immobilized liquid membranes. Sep. Sci. Technol..

[58] Sanip S.M., Ismail A.F., Goh P.S., Soga T., Tanemura M., Yasuhiko H. (2011). Gas separation properties of functionalized carbon nanotubes mixed matrix membranes. Sep. Pur. Technol..

[59] Bastani D., Esmaeili N., Asadollahi M. (2013). Polymeric mixed matrix membranes containing zeolites as a filler for gas separation applications: A review. J. Ind. Eng. Chem..

[60] Karimi M.B., Khanbabaei G., Sadeghi G.M.M. (2017). Vegetable oil-based polyurethane membrane for gas separation. J. Membr. Sci..

[61] Drioli E., Criscuoli A., Curcio E. (2011). Membrane Contactors: Fundamentals, Applications and Potentialities.

[62] Esquiroz-Molina A., Georgaki S., Stuetz R., Jefferson B., McAdam E.J. (2013). Influence of pH on gas phase controlled mass transfer in a membrane contactor for hydrogen sulphide absorption. J. Membr. Sci..

[63] Uhlmann D., Smart S., da Costa J.C.D. (2011). H_2_S stability and separation performance of cobalt oxide silica membranes. J. Membr. Sci..

[64] Tilahun I., Bayrakdar A., Sahinkaya E., Çalli B. (2017). Performance of polydimethylsiloxane membrane contactor process for selective hydrogen sulfide removal from biogas. Waste Manag..

[65] Tilahun I., Sahinkaya E., Çalli B. (2018). A hybrid membrane gas absorption and bio-oxidation process for the removal of hydrogen sulfide from biogas. Int. Biodeterior. Biodegrad..

[66] Shah M.S., Tsapatsis M., Siepmann J.I. (2017). Hydrogen Sulfide Capture: From Absorption in Polar Liquids to Oxide, Zeolite, and Metal-Organic Framework Adsorbents and Membranes. Chem. Rev..

[67] Atlaskin A.A., Kryuchkov S.S., Yanbikov N.R., Smorodin K.A., Petukhov A.N., Trubyanov M.M., Vorotyntsev V.M., Vorotyntsev I.V. (2020). Comprehensive experimental study of acid gases removal process by membrane-assisted gas absorption using imidazolium ionic liquids solutions absorbent. Sep. Pur. Technol..

[68] Cuevasanta E., Denicola A., Alvarez B., Möller M.N. (2012). Solubility and Permeation of Hydrogen Sulfide in Lipid Membranes. PLoS ONE.

[69] Mansourizadeh A., Ismail A.F. (2009). Hollow fiber gas-liquid membrane contactors for acid gas capture: A review. J. Hazard. Mater..

[70] Abdelnabi M.M., Cordova K.E., Abdulazeez I., Alloush A.M., Al-Maythalony B.A., Mankour Y., Alhooshani K., Saleh T.A., al Hamouz O.C.S. (2020). A Novel Porous Organic Polymer for the Concurrent and Selective Removal of Hydrogen Sulfide and Carbon Dioxide from Natural Gas Streams. ACS Appl. Mater. Interfaces.

[71] Zhang L., de Schryver P., de Gusseme B., de Muynck W., Boon N., Verstraete W. (2008). Chemical and biological technologies for hydrogen sulfide emission control in sewer systems: A review. Water Res..

[72] Ghimpusan M., Nechifor G., Din I.S., Nechifor A.C., Passeri P. (2016). Application of Hollow Fibre Membrane Bioreactor Instead of Granular Activated Carbon Filtration for Treatment of Wastewater from Car Dismantler Activity. Mater. Plast..

[73] Din I.S., Cimbru A.M., Rikabi A.A.K.K., Tanczos S.K., Ticu S., Nechifor G. (2018). Iono-molecular Separation with Composite Membranes VI. Nitro-phenol separation through sulfonated polyether ether ketone on capillary polypropylene membranes. Rev. Chim..

[74] Nafliu I.M., Al-Ani H.N.A., Grosu A.R., Albu P.C., Nechifor G. (2019). Iono-molecular separation with composite membranes. VIII. Recuperative aluminium ions separation on capilary polypropylene S-EPDM composite membranes. Mater. Plast..

[75] Shaw J.A. (1940). Rapid determination of hydrogen sulfide and mercaptan sulfur. In gases and in aqueous solutions. Anal. Chem..

[76] Paré J.P. (1966). A new tape reagent for the determination of hydrogen sulfide in air. J. Air Pollut. Control Assoc..

[77] Natusch D.F.S., Sewell J.R., Tanner R.L. (1974). Determination of hydrogen sulfide in air—An assessment of impregnated paper tape methods. Anal. Chem..

[78] Windholz M. (1976). Hydrogen sulfide. Merck Index.

[79] Van der Bruggen B., Vandecasteele C., van Gestel T., Doyen W., Leysen R. (2003). A review of pressure-driven membrane processes in wastewater treatment and drinking water production. Environ. Prog..

[80] Strathmann H., Giorno L., Drioli E. (2011). Introduction to Membrane Science and Technology.

[81] Damuchali A.M., Guo H. (2019). Evaluation of a field olfactometer in odour concentration measurement. J. Biosyst. Eng..

[82] Gallego E., Roca F.J., Perales J.F., Sanchez G., Esplugas P. (2012). Characterization and determination of the odorous charge in the indoor air of a waste treatment facility through the evaluation of volatile organic compounds (VOCs) using TD-GC/MS. Waste Manag..

[83] Ghimpusan M., Nechifor G., Nechifor A.C., Dima S.O., Passeri P. (2017). Case studies on the physical-chemical parameters’ variation during three different purification approaches destined to treat wastewaters from food industry. J. Environ. Manag..

[84] Grosu A.R., Nafliu I.M., Din I.S., Cimbru A.M., Nechifor G. (2020). Neutralization with simultaneous separation of aluminum and copper ions from condensed water through capillary polypropylene and cellulose. UPB Sci. Bull. Ser. B Chem. Mater. Sci..

[85] Allen J.T., de Lauretis T., Heath S. (1980). The Industrial Context of Film Technology: Standardisation and Patents. The Cinematic Apparatus.

[86] Cilibertoa E., Gemmellaroa P., Iannusoa V., la Delfaa S., Ursoa R.G., Viscuso E. (2013). Characterization and weathering of motion-picture films with support of cellulose nitrate, cellulose acetate and polyester. Procedia Chem..

[87] Ahmad I.R., Cane D., Townsend J.H., Triana C., Mazzei L., Curran K. (2020). Are we overestimating the permanence of cellulose triacetate cinematographic films? A mathematical model for the vinegar syndrome. Polym. Degrad. Stab..

[88] Camera Deputaților, Romania. http://www.cdep.ro/interpel/2017/r573B.pdf.

